# Offline Memos for Online Teaching: A Collective Response to *The Manifesto for Teaching Online* (Bayne et al. 2020)

**DOI:** 10.1007/s42438-022-00286-4

**Published:** 2022-01-27

**Authors:** Anna Blumsztajn, Wiebe Koopal, Pia Rojahn, Hans Schildermans, Bianca Thoilliez, Joris Vlieghe, Kai Wortmann

**Affiliations:** 1 University of Gdańsk, Gdańsk, Poland; 2 KU Leuven, Leuven, Belgium; 3 University of Wuppertal, Wuppertal, Germany; 4 University of Vienna, Vienna, Austria; 5 Universidad Autónoma de Madrid, Madrid, Spain; 6 University of Tübingen, Tübingen, Germany

**Keywords:** Higher education, Digitization, Teaching, Public–private distinction, Educational design, Ecologies of teaching/learning, Attention

## From Reading a Manifesto to Writing Memos

This paper is the reflection of the proceedings of a live colloquium held by seven educational researchers in Madrid in August 2021. Point of departure of this colloquium was a shared concern for the ascendance of online teaching as the privileged mode of teaching in higher education. This concern, which had been influenced significantly by our various experiences during the Covid-19 crisis, was recently articulated in an outspokenly affirmative and positive manner by a group of digital education scholars in *The Manifesto for Teaching Online* (Bayne et al. 2020). In contrast, however, our own discussions showed that, whereas most of us deeply appreciated *The Manifesto’s* efforts to think through the possibility of genuinely pedagogical approaches to online teaching, we still found fault with its glaring omission of substantial arguments for online modalities of higher education *as such*. *The Manifesto* mostly works on the assumption that online teaching can always be of added value vis-à-vis offline teaching, yet often ‘forgets’ balancing out these assets against the qualities of offline teaching that risk getting lost.

At the same time, our discussions during the colloquium revealed significant intellectual divergences between the different stances that the participants took with regard to how these particular qualities can be understood and conceptualized. After we failed to come to a shared, unified statement—a manifesto—it was decided that, instead of regarding this as a deficit, we could opt for the alternative idea of a series of short memos, in the style of Italo Calvino’s *Six Memos for the Next Millennium* (1988). Inspired by different intellectual stances and interests, yet still in view of a common concern for online, face-to-face higher education, these memos would have to form a disparately resonant ‘call to attention’ for the ascending online modalities and their discourses.

Accordingly, our paper is divided into seven short sections, each of which forms a memo that expands on one particular quality of higher education—a quality which, while mostly also addressed in *The Manifesto*, we believe still remains more authentically connected to offline modes of education. The seven qualities under concern are the following: publicness, world-disclosure, friction, joint attention, rhythm, secrecy, metastability. We believe that each of these qualities is *already* present in offline teaching, even though they are not always noticed and valued, and that the realization of each of these qualities requires specific care and attention in the design of practices of online teaching. In that sense, we do not so much wish to criticize *The Manifesto*, as to point toward something that risks being forgotten.

Finally, each memo discusses its quality against the backdrop of a phenomenological description of a real/fictive experience to prevent our analyses from becoming unduly abstract. Except for this shared element, however, the memos do not have any uniform discursive style. Perhaps this perspectival diversity would only add to the strength of our discourse, doing justice to the multiplicity of nuances and values that give shape to the concerns at stake. At the same time, while thus the memos could be read as individual contributions, we have opted to keep them anonymous, viz. to maintain them as interconnected pieces of a *collective* work. After all, since they are the result of several days of common interaction, their authorship is far from individual.

## Memo 1: Publicness

*Sam wakes up, eats breakfast with her flatmates in the shared kitchen. She goes back to her room, sits down on her bed, opening her laptop. Still in her pyjamas, she checks some YouTube videos and simultaneously chats with some friends. Then she notices that it’s time to log into her first university seminar. Still sitting on her bed, she logs in. Because she didn’t have the time to take a shower yet, she decides to turn her camera off. While listening to the teacher’s introduction, she notices a pile of clothing that she meant to organize before. So, while listening, she organizes her room. When she’s really concentrated on the seminar, a flatmate enters her room and asks her if she could buy some milk later. After this interruption, she tries to follow the discussion again. Yet, the talk with her flatmate reminds her of the grocery shopping. She quickly makes a list of things. Suddenly, her phone rings. Her mum calls to ask if she’ll join a family gathering on Sunday. After that, she really tries to focus on the seminar discussion again. However, the seminar is nearly over. She decides to take her laptop to the kitchen and prepare some coffee…*

University teaching is public. It needs a clear separation from the private sphere, especially from the everyday necessities. The physical act of leaving your home to enter the university campus symbolizes the conscious mental act of leaving your private thoughts to engage with the thinking of others. Students and teachers prepare themselves before entering a seminar room and usually accept certain common rules when they consciously decide to participate in a seminar (e.g., phones turned off, no eating, etc.).

While reading the chapter on ‘Face, Space and Place’ in *The Manifesto for Teaching Online* (Bayne et al. 2020), it seemed surprising that the authors did not address how much the private infiltrates the shared educational space during online seminars. Although *The Manifesto*’s authors notice that educational space can be affected by the political situation of their students (Bayne et al. 2020: Ch. 18), they do not raise the question what it means that the private enters into the public sphere of university education.

Hannah Arendt underlines the importance of the distinction between the private and the public. She describes the private sphere as the place of necessity, where we take care of our physical well-being and where we rest from engagement in the public realm (Arendt 1958: 32-40). The private is the sphere of exclusiveness. ‘Here we choose those with whom we wish to spend our lives … those we love; and our choice is guided not by likeness or qualities shared by a group of people – it is not guided, indeed, by any objective standards or rules.’ (Arendt 1959: 52)

In contrast, the public is the sphere of equality (Arendt 1959: 51), where individuals grant each other the same rights to engage in discussions among equals. It is also the space of plurality, in which you learn to deal with different people’s positions, even though you would not choose to share your private place with them. Joining a university seminar confronts you with the plurality of the world—physically and mentally. Because of that, it offers the ideal environment to train ‘enlarged thinking’—which is the basis for judgement (Kant 1790; Arendt 1982). University seminars not only offer the opportunity but force you to deal with different perspectives that you would not engage with if you stayed at home.

## Memo 2: World-Disclosure

*Suddenly and out of the blue her teaching is interrupted. The colleague that runs an event after her class is finished - a prominent guest is expected to give a talk in the same auditorium - has entered ten minutes before the end of her class so as to force her and the students to leave the room. She is very upset and can’t help starting to rail at the intruder, using a language that is not so polite. After this, she is not able to find the right tone to continue her teaching.*

*The Manifesto for Teaching Online* (Bayne et al. 2020) could be regarded first and foremost as a manifesto for teaching. It makes strong claims about our educational present and future and puts important dimensions of teaching on display—as in the original meaning of ‘to manifest’ [to prove by direct evidence, i.e., by things one can hold (*festus*) in one’s hand (*manus*)]. Hence, what is declared in this text draws its persuasiveness from what we may and may not directly experience.

What *The Manifesto* decisively shows is that conferencing technologies we have massively relied on lately cannot substitute face-to-face interaction. It claims, on the contrary, that good online teaching needs to tap into the particular potentials intrinsic to digital technologies, and that teachers should not be regarded as the executioners of plans devised by (EdTech) specialists. Instead, online teachers are to be deeply involved in the development of all aspects of their teaching.

All this sounds persuasive. It remains unconvincing; however, why we would want to do away with face-to-face teaching in the first place. Until made manifest otherwise, face-to-face encounters seem vital for some of the basic operations of teaching.

Teaching means *that one discloses one’s love for the world*. Typically, one does this by putting on display care for the thing one teaches about. It matters that students are allowed to witness the precision with which a theorem is constructed on the blackboard, the pleasure and ardor with which the cooking instructor explains how to prepare an omelet, the carefulness and respect with which a teacher puts her textbooks away, the anger when teaching is disturbed or jeopardized as in the vignette. In such details, subject matter comes to matter as a thing worthy of study and care.

Teaching also means showing to newcomers: ‘this is our world’ (Arendt 1959). Students experience that something more is at stake than self-development and individual growth: *they become truly interested in a world shared with others*. That is why students literally *go* to school and university: they leave behind the safety and comfort of the home to spend time elsewhere, in the presence of other people which they haven’t chosen and which they do not necessarily like. Hence, students can experience that education is not only a learning process aimed at them as singular individuals, but *that they are addressed as representatives of a new generation*.

Whether online teaching can *manifest*ly do the same (or better) is an issue worth researching further.

## Memo 3: Friction

*An online lecture about theories of discrimination. The teacher is lecturing, analyzing some quotes from relevant authors, sharing them on the screen. One of the quotes sparks a discussion between students, first in the lecture’s chat box, then in their messenger group. Soon the quarrel turns into a series of interpellations and identity claims. The teacher finishes her lecture half an hour earlier in order to leave time for discussion. But when she asks for live questions or comments, there are none. The teacher is left with answering individual questions and comments in the chat box, but some of the students have already left and are not there to read them.*

The authors of *The Manifesto for Teaching Online* (Bayne et al. 2020) rightly claim that the mediating technologies of online teaching can be empowering for some, allowing them to engage in the education process more easily than in an offline setting, where shyness toward peers, and submission to group hierarchies, may render participation difficult. The authors also frequently refer to the fluidity of the online educational process, the de-incarnation made possible by virtual technologies (as with avatars for instance) as an educationally emancipating feature, fostering creativity.

While there is no reason to question this account, it should bring our attention to the facility created by an online teaching situation and inspire us to analyze and problematize it more deeply. In fact, while we can notice that some students engage more easily in an online setting, we should look closer at the kind of involvement and communication happening there and inquire where does the easiness come from and what does it do to the educational process?

Online education context has its participants' presence mediated by interface technology that allows each of them to make constant individual decisions as to the quality, scale and intensity of their being there. Who (what part of me, what identity traits?) is participating in the educational situation, and to what extent, is a matter of constant redefinition according to individual judgement. As the vignette tries to illustrate, technological mediation and virtuality of presence make it easy to engage. But it also makes it easy to withdraw, to exclude, to be bold without consequences; to speak to others, without talking with them, without responsibility for consequences.

Face-to-face teaching involves a multitude of tensions associated with being bound to the direct experience of others, and to being experienced by them, in a given time and space. Offline education with physical co-presence produces group dynamics and processes, a social materiality that has its own bumps and roughness. Those fundamental social frictions are an integral part of education. Mediation created by an online setting results in increased individual agency, allows escaping an always demanding social materiality, and stands in the way of difficulties related to coming together and educating one another.

## Memo 4: Joint Attention

*At night, in desolate and dark university buildings during lockdown, students gather in an empty seminar room. They lower the projection screen, turn on the beamer, rearrange desks and chairs, and sit down to watch a movie together. In this small bubble, gathered around the screen, they rediscover the power of collectivity on their ability of paying attention.*

The authors of *The Manifesto for Teaching Online* (Bayne et al. 2020) claim that co-presence is overstated and that contact is possible also in online environments. This seems to imply that co-presence is not an aspect of teaching online, or at least not its essential aspect. However, considering that educational practices are always already technologically mediated (be it by pencil and paper or by PowerPoint presentation), it can be argued that teaching online stages *its own mode* of co-presence, even if this co-presence is not (entirely) embodied. This requires careful thinking about the ways in which online learning environments are being designed, and how these designs help or hinder the coming into being of co-presence.

Such thinking implies to take seriously the ‘ecological agency’ of educational practices, including those that take place offline just as much as those organized online. Addressing ecological agency of educational practices means to attend to the ways in which educational practices create environments for learning and thinking together, for teaching, for paying attention to things of interest, for raising questions and trying a response (Citton 2019; Rasmi 2021).

Ecological agency of educational practices enacts moments of joint attention. Citton (2019: 25) defines joint attention as ‘the situations in which the participants’ attentions affect each other in real time and in mutual interactions’. Paying attention is, therefore, not something that an engaged student chooses to do, while the lazy students neglect their duty. Neither are attentive students the prerogative of the outstanding and inspiring teacher. Rather, attention is the effect of a carefully crafted situation that involves other bodies held together in specific architectures, holding their breath while feeling the attentive presence of others. Therefore, although it is possible to think educational practices without co-presence, it seems that joint attention always involves some kind of being together. Attention as ecological agency presupposes an environmental arrangement that includes bodies, architectures, things, technologies, and tools.

Claiming that co-presence is overstated, therefore, seems to be a risky statement. It reduces subject matter to commodified knowledge contents that can be exchanged via online platforms. Taking seriously the ecological agency of educational practices in creating moments of joint attention means indeed to take seriously the event-like character of lecturing itself. Lecturing, in that sense, is an event that is dependent upon multiple ecological factors that enact a specific mode of belonging, a way of paying attention. This form of participation should not be understood strictly as active involvement, but entails elements of bodily presence and environmental factors that are conducive to joint attention. It is perhaps this kind of joint attention the students in the vignette were looking for.

## Memo 5: Rhythm

*Two people are talking simultaneously. They notice and stop talking. Silence. Then, again simultaneously: Please, go ahead! Silence. After a while that felt like ages, as if they heard a starter’s gun, they both continue…* (D)

Why does this happen online so much more frequently than offline? And why is it a source of exhaustion in the first case and laughter in the latter? Is it maybe because of the groove created by face-to-face conversation, a groove that, because of the delay, is much harder (maybe even impossible) to achieve online? To act in concert with each other, as Arendt puts it, requires timing—a feeling for the entry of one’s voice—and timing gets harder with delay. (C)

In Whitehead’s (1929) view of life as a continuing process, education is of particularly rhythmic character. This emphasizes the aesthetic dimension of education in general and arguably of seminar discussions. Their temporality requires an immediacy to get the timing right, without which the concert of collective discussion turns into a cacophony. (B)

Imagine the scene described in the vignette happening in a seminar discussion: Wouldn’t that be hilarious? It would signal a sense of urgency, of the two protagonists intensely feeling the need to share something with the group. Online, the same thing happens not because of a *specific* groove, but because of the absence of *any*. (A)

(A) The playing with musical metaphors in this piece owes a lot to the works of musicologist Peter Wicke. We strongly believe that the transfer of the implications from categories used for analyzing pop music, such as groove, performance, and sound (e.g., Wicke 2008), could very much inspire the analysis of education.

(B) We acknowledge that Whitehead uses the term rhythm to describe a circular educational process of three stages—romance, precision, and generalization. He describes the possibility of different scales of the three stages; however, he does not write about rhythm within concrete educational interaction (Whitehead 1929).

(C) Again, we must acknowledge somewhat of a stretch in my borrowing of terms. The metaphor of acting in concert with each other for Arendt (1958: 200) belongs to the realm of political life to which for her education clearly does not belong. Nevertheless, it could be fruitful to explore this notion further also in relation to education since for Arendt it is an answer to the – arguably also prevalent educational question—of how to combine equality and plurality (see Honig 1995; Mori 2003).

(D) This situation was inspired by the video ‘A Conference Call in Real Life’ by sketch duo Tripp and Tyler.^1^ It transfers some of the technical problems of video conferences into a real-life setting and thereby intensifies the social awkwardness they create. This in turn makes the business meeting impossible to be about actual business. The same might be said for education. We thank Joris Vlieghe for showing us the video during our stay in Madrid.

## Memo 6: Secrecy

*When the session finished a link to the video was automatically generated. Susanne clicked on it. She got redirected to the Faculty’s YouTube page.*

*- What did I click on?*

*She clicked the back button. Then she clicked on the autogenerated link again. Susanne did not know the channel she was lecturing on was mistakenly connected with the YouTube channel of the faculty. But there it is. The whole thing was being streamed online, meaning all subscribers of the channel got a message that an ‘event’ was being launched. Once the event is finished, the recording gets autosaved and suggested as ‘newest video’. This was a late Friday class, so nobody from the media department is reachable. The video ends up staying there for the whole weekend, with comments open to everyone. Comments about how messy her hair was, her non-native English accent, how boring this class is, alleged former students sharing notes and exams from previous years, mockery of her students’ questions during the class…*

Closing a classroom door opens opportunities to retreat from the public eye. Going online is exactly the opposite. Teaching always implies some degree of exposure (Standish 2016). When streamed online, that exposure turns into a potentially massively distributed, consumed, and discarded commodity.

Lecture halls and seminar rooms are spaces where private self-recreations and public solidarity debates can be practiced (Rorty 1993). To secure this, teachers and students require safety provided by privacy of the classroom. Learning needs freedom and lightness that arrive with not being reminded, recordable, forever available. Privacy enables teachers and students to take risks without being afraid of misplacements, decontextualization, or being perceived as (in)appropriate.

Arendt (1961) referred to this when commenting on the excessive exposure experienced by children from famous households.

Everything that lives, not vegetative life alone, emerges from darkness and, however strong its natural tendency to thrust itself into the light, it nevertheless needs the security of darkness to grow at all. This may indeed be the reason that children of famous parents so often turn out badly. Fame penetrates the four walls, invades their private space, bringing with it, especially in present-day conditions, the merciless glare of the public realm, which floods everything in the private lives of those concerned, so that the children no longer have a place of security where they can grow. (Arendt 1961: 183)

Social networks have enabled and/or accustomed everyone to expose their lives, and lives of their children, to the public eye. Therefore, Arendt’s analysis is now a much more common experience for many children. Something similar happens when classroom walls become transparent via lecture recordings. A classroom is not meant to be as safe as a home, but it is still not a fully open public space. Classroom is a transitioning place, where missteps can take place safely.

Some teachers shared their classes on the web way before the Covid-19 pandemic turned us all into precarious EduTubers. If online teaching is ever turned into ‘the privileged mode’ for teaching, their experience is telling of some situations we could find ourselves in. For example, Ernesto Castro^2^ and Jordan B. Peterson^3^ have YouTube channels started as simple recordings of their face-to-face lectures, followed by other, more elaborate sorts of content. Starting from the oldest lecturing recordings and browsing toward more recent ones, some questions emerge. Who are they really talking to, their students or other YouTube viewers? Are students being turned into walk-on actors of a show? Does uploading lectures on YouTube create a teacher-centered situation? How many students are acting more like spectators rather than actual students, because of the fact their questions will be streamed, recorded, and shown to the outside-the-classroom-world?

For the potentialities of teaching to unfold, some degree of privacy is needed. That does not mean that teachers should not be responsible for their work. Yet to make teaching and learning happen, teachers and students need to withdraw from the public scene.

## Memo 7: Metastability

*Huddled up before the open door, whose porch we haven’t yet dared to cross, we start trickling into the auditorium, our lunchtime chatter slowly dissipating. We take our places—usually the same as ever, as if they had been assigned—and get out laptops, notepads, writing gear, water bottles. The next few minutes, the auditorium remains vaguely virginal, with most of us vacuously stare at one another, or at the algebraic formulas scribbled on the blackboard; in some corners, chatter resumes. But then, lo… through the door-opening enters the professor, six minutes late, quite ‘on time’. He pays no attention to us, walks over to his desk, and begins unpacking: his worn-out textbook is laid open, another empty blackboard is scrolled over the algebraic formulas (which briefly fascinate him), and he connects his laptop to the beamer—with the usual grumbling. Suddenly, a light-flash: the beamer is projecting; first some e-mail that we’re not supposed to see, then, after more grumbling, today’s presentation. We mechanically read:* Rousseau and Thing-centered Education*; oh boy… Immediately Facebook pages open up. Yet more than the beamer, which now projects the opening still of a (black and white!) YouTube video, our attention is caught by the beaming of the professor’s eyes. He has lifted them towards us. For just about a minute, he watches intently, then slightly nods (and sighs?), before striding towards the door, which he solemnly closes. We gaze at him, at the whole unfolding spectacle, and realize: the show is on, whether we like it or not. All of the world’s chatter and turbulence are shut out now (there are even no windows to suggest its existence), and for the next two hours* this *will make a world; a hell perhaps, for some, but then, we’ll have to live in it…*

What this undoubtedly recognizable scene depicts, is what I propose to call (after Simondon 2005) the ‘metastability’ so characteristic of much offline education. While for plausible reasons digital/online education prides itself on being open to a much higher degree of ‘flexibility’ and ‘multimodality,’ I believe that the vignette puts this claim somewhat into perspective. After all, does the scene depicted here not represent a veritable *tableau vivant*, containing within its seemingly static space an abundance of more or less subtle and intersecting, movements? And are these dynamics at the same time not also enacted in quite a diverse array of educational modalities—bodies, auditorium, paper, screens, blackboards, text, film—in a way that renders their modal differences immediately tangible?

Asking these questions, we do not suggest that online education has no real and precious merits. Quite the contrary, it certainly heightens our awareness of the educational seductions of flexibility and multiplicity. Yet what the vignette inversely also hints at is the need which these qualities paradoxically have for a *certain*, palpable closure and uniformity, which we call ‘metastability’. To be sure, this has nothing to do with the stability of inert substance [which *The Manifesto for Teaching Online* (Bayne et al. 2020) mostly alludes to when targeting offline education]: it is *meta* because it regards the educational event as a *threshold*, a provisionally separate *and* stable spacetime which binds together a purposeful selection of reality’s dynamics and means. As the auditorium scene shows, this metastability still lives off the irreducible flexibility and multiplicity of reality’s forces, but equally restrains these, twisting them to ‘reflect’ upon themselves, rather than dissipating in an inoperably chaos. This shared restrain, which indeed induces a certain stability or even rigidity, is *not* a weakness, it is a strength, a condition even for making the aforementioned forces *more* fecund than they would be in a state of absolute flexibility.

## Coming to a Closure

Despite the variety of the qualities of offline teaching to which we have tried to draw attention, we believe they all attest to a dimension of our educational practices that is shared among the diverse memos, namely the *ecology* of (higher education) teaching. Education does not happen in a vacuum, but there are always multiple, contextual, and environmental aspects or forces (architectures, technologies, shared visual and acoustic spaces, etc.) that situate our practices according to their own specific agencies. It is these forces that need to be taken into account while discussing offline and online teaching and that put specific demands on the design of practices of teaching online (like the force of gravitation puts a specific demand on the design of a building).

We suggest to grasp these demands as issuing from a set of obligations. Stengers (2010) explains that an obligation is not a normative principle or rule of conduct (for ‘good’ online teaching in this case). Rather, speaking in terms of an obligation pays tribute to the fact that our thoughts depend on the existence of something that ‘obliges’ us, makes us think. It is a moment of hesitation in which what tends to be forgotten starts to insist on our established habits of thought. It is these obligations that make us hesitate in front of the mobilization to go online that we want to specify.

### Obligation 1

**We cannot not escape from positioning ourselves in relation to the tension between the public and the private sphere**. Some memos stress the necessity of private closure and safety (6), of a certain intimacy that structures the flow of our spoken interactions (5), and of separate spacetimes that allow for metastability (7). However, others emphasize the profound public character and the importance of the presence of strangeness typical to school spaces, i.e., places that are not always immune, comfortable, or safe (1, 2, 3, 4).

### Obligation 2

**We are not fully in control over the meaning of our practices, as bodies, things, tools, and architectural arrangements make us act and experience in specific ways**. There is no experience of friction without exposure and the group dynamics of social materiality (3), no world-disclosure without gesticulation (2), no student engagement or joint attention without ecological agency (4). Neither is there any common thought nor equality without more or less secluded rules and rituals (1, 6), no educational rhythm without the right, i.e., non-delayed timing of our conversations (5), and no temporal stabilization that is necessary for teaching without the constellation of nested bodies and things in the auditorium (7).

### Obligation 3

**Certain offline educational practices are important enough to keep, but are as such also very much dependent on a non-reactionary, well-reflected and situated sustenance and care**. This collection stresses the *possibility* to keep defending offline practices of higher education as the privileged mode. Nevertheless, our divergent accounts of the diverse characteristics connected to that possible privilege also show that these cannot be glued together in one coherent picture. In the end, retrospectively, such coherence could never have been our ambition.

## Footnotes

1 See https://youtu.be/TwBiBWDep5I. Accessed 3 December 2021.

2 See https://www.youtube.com/channel/UCd3FJ4k849ZtZXUL3MsXyOA. Accessed 3 December 2021.

3 See https://www.youtube.com/channel/UCL_f53ZEJxp8TtlOkHwMV9Q. Accessed 3 December 2021.
